# Brain derived neurotrophic factor/tropomyosin related kinase B signaling impacts diaphragm neuromuscular transmission in a novel rat chemogenetic model

**DOI:** 10.3389/fncel.2022.1025463

**Published:** 2022-10-28

**Authors:** Matthew J. Fogarty, Obaid U. Khurram, Carlos B. Mantilla, Gary C. Sieck

**Affiliations:** ^1^Department of Physiology and Biomedical Engineering, Mayo Clinic, Rochester, MN, United States; ^2^Department of Anesthesiology and Perioperative Medicine, Mayo Clinic, Rochester, MN, United States

**Keywords:** diaphragm muscle, neuromuscular junction, neuromuscular transmission failure, neurotrophins, genetic models

## Abstract

The neuromuscular junction (NMJ) mediates neural control of skeletal muscle fibers. Neurotrophic signaling, specifically brain derived neurotrophic factor (BDNF) acting through its high-affinity tropomyosin related kinase B (TrkB) receptor is known to improve neuromuscular transmission. BDNF/TrkB signaling also maintains the integrity of antero- and retrograde communication between the motor neuron soma, its distal axons and pre-synaptic terminals and influences neuromuscular transmission. In this study, we employed a novel rat chemogenetic mutation (*TrkB*^*F*616^), in which a 1-naphthylmethyl phosphoprotein phosphatase 1 (1NMPP1) sensitive knock-in allele allowed specific, rapid and sustained inhibition of TrkB kinase activity. In adult female and male *TrkB*^*F*616^ rats, treatment with either 1NMPP1 (TrkB kinase inhibition) or DMSO (vehicle) was administered in drinking water for 14 days. To assess the extent of neuromuscular transmission failure (NMTF), diaphragm muscle isometric force evoked by nerve stimulation at 40 Hz (330 ms duration trains repeated each s) was compared to isometric forces evoked by superimposed direct muscle stimulation (every 15 s). Chronic TrkB kinase inhibition (1NMPP1 group) markedly worsened NMTF compared to vehicle controls. Acute BDNF treatment did not rescue NMTF in the 1NMPP1 group. Chronic TrkB kinase inhibition did not affect the apposition of pre-synaptic terminals (labeled with synaptophysin) and post-synaptic endplates (labeled with α-Bungarotoxin) at diaphragm NMJs. We conclude that inhibition of BDNF/TrkB signaling in *TrkB*^*F*616^ rats disrupts diaphragm neuromuscular transmission in a similar manner to *TrkB*^*F*616*A*^ mice, likely *via* a pre-synaptic mechanism independent of axonal branch point failure.

## Introduction

In mammals, the diaphragm muscle (DIAm) is the principal muscle responsible for breathing by generating negative intrathoracic pressure necessary for driving airflow into the lungs (Fogarty and Sieck, 2019b). The DIAm also contributes to the generation of positive abdominal pressure essential for expulsive behaviors including defecation, coughing, and sneezing (Sieck and Fournier, 1989; Fogarty and Sieck, 2019a). Phrenic motor neurons (PhMNs) located in the cervical spinal cord innervate the DIAm, with an orderly recruitment of motor units underpinning different DIAm motor behaviors (Sieck and Fournier, 1989; Fogarty and Sieck, 2019a). In general, smaller PhMNs with slower axonal conduction velocities are recruited first whereas larger PhMNs with faster axonal conduction velocities are recruited later (Dick et al., 1987). Different DIAm motor unit types are distinguished by their mechanical and fatigue properties in accordance with their fiber type composition, with slow (type S) units comprising fatigue resistant type I fibers, fast fatigue resistant (type FR) comprising type IIa fibers, and fast fatigable (type FF) comprising type IIx and/or IIb fibers (Sieck et al., 1989; Fogarty and Sieck, 2019b).

Neural control of the DIAm is mediated *via* neuromuscular junctions (NMJs) consisting of pre-synaptic axon terminals and post-synaptic endplates. A failure in neuromuscular transmission has deleterious effects on DIAm performance, especially if it occurs during breathing as well as airway defense and other expulsive behaviors (Fogarty and Sieck, 2019b). Previously, we demonstrated significant neuromuscular transmission failure (NMTF) in the rodent DIAm during repetitive higher-frequency stimulation (i.e., 40 and 75 Hz) of the phrenic nerve (Kuei et al., 1990; Johnson and Sieck, 1993; Fogarty et al., 2019, 2020a). In addition, the incidence of NMTF is greater in type FF motor units (type IIx and/or IIb fibers) stimulated at higher frequencies (i.e., >40 Hz) (Krnjevic and Miledi, 1958, 1959; Johnson and Sieck, 1993). Conditions such as aging, spasticity, and muscular dystrophy can exacerbate DIAm NMTF (Personius and Sawyer, 2006; Greising et al., 2015a; Fogarty et al., 2019, 2020a), whereas neurotrophic signaling improves neuromuscular transmission (Mantilla et al., 2004, 2014; Ermilov et al., 2007; Mantilla and Ermilov, 2012). Notably, NMTF can arise in the absence of overt morphological perturbation at NMJs, likely due to action potential propagation failures at axonal bifurcations along the phrenic nerve (Sieck and Prakash, 1995) or impaired recycling of synaptic vesicles following repetitive activation (Rowley et al., 2007). The extent of NMTF worsens under conditions of expanded motor unit innervation ratio (i.e., number of muscle fibers innervated by a motor neuron) such as in the case of developmental spasticity in the spa mouse, where a reduced number of PhMNs (Fogarty et al., 2020a) innervate the normal overall number of DIAm fibers. In rats, age-related loss of PhMNs (Fogarty et al., 2018) increases the motor unit innervation ratio resulting in a worsening NMTF (Fogarty et al., 2019).

Neurotrophins exert both pre- and post-synaptic effects at NMJs throughout the lifespan, with altered neurotrophic signaling suspected to underlie various neuromotor disorders (Funakoshi et al., 1995; Sieck and Mantilla, 2009; Garcia et al., 2010a,b; Jablonka et al., 2014). Specifically, brain derived neurotrophic factor (BDNF) signaling *via* its high-affinity full-length tropomyosin receptor kinase (TrkB_*FL*_) mediates synaptic plasticity at the NMJ, likely *via* phosphorylation of the transcription factor CREB (Finkbeiner et al., 1997; Esvald et al., 2020). The potent developmental effects of altered BDNF/TrkB signaling at NMJs was clearly demonstrated in transgenic TrkB_*FL*_ knockout mice (Garcia et al., 2010b). Notably, TrkB_*FL*_ knockout mice do not survive postnatally. Accordingly, to examine the effects of altered BDNF/TrkB signaling in adult mice, we previously employed a chemogenetic *TrkB*^*F*616*A*^ mouse model (Chen et al., 2005) that expresses knock-in alleles, which permit rapid, selective, and reversible inhibition of TrkB_*FL*_ kinase activity *via* exposure to the kinase inhibitor (TrkB kinase inhibited–TKI), 1-Naphthylmethyl phosphoprotein phosphatase 1 (1NMPP1) (Benjamin et al., 2003; Wang et al., 2003). In adult *TrkB*^*F*616*A*^ mice treated with 1NMPP1 for 7 days (TKI mice), we demonstrated substantial worsening of DIAm NMTF and modest retraction of pre-synaptic terminals at NMJs (Mantilla et al., 2014). We also observed that acute *ex vivo* 1NMPP1-induced TrkB kinase inhibition DIAm NMTF worsened (Mantilla and Ermilov, 2012). Notably, the effects of 7-day TKI on DIAm NMTF (Greising et al., 2015b) were completely reversed by 7-day vehicle treatment (Mantilla et al., 2014).

In the present study, we assessed NMTF following chemogenetic inhibition of BDNF/TrkB signaling in *TrkB*^*F*616^ TKI rats. We hypothesized that in *TrkB*^*F*616^ TKI rats, chronic 1NMPP1-induced inhibition of TrkB kinase will worsen DIAm NMTF.

## Materials and methods

### Animals and anesthesia

This study was approved by the Institutional Animal Care and Use Committee (IACUC) and all procedures were performed in accordance with American Physiological Society’s *Guiding Principles in the Care and Use of Vertebrate Animals in Research and Training*. Adult (4 months of age) female and male *TrkB*^*F*616^ rats (*n* = 8 per sex) were used to selectively, rapidly, and reversibly inhibit TrkB kinase activity following oral 1NMPP1 treatment (25 μM in drinking water). *TrkB*^*F*616^ rats were generated on a Sprague Dawley hybrid genetic background and were genetically modified to stably harbor a knock-in mutation that results in inhibition of TrkB kinase activity only in the presence of PP1 derivatives such as 1NMPP1, in a manner similar to the *TrkB*^*F*616*A*^ mouse (Chen et al., 2005), that we have used in prior studies (Mantilla and Ermilov, 2012; Mantilla et al., 2014; Greising et al., 2015a,b). All rats were individually housed and maintained on a 12-h light-dark schedule under specific pathogen-free conditions with *ad libitum* access to food and water.

**FIGURE 1:**
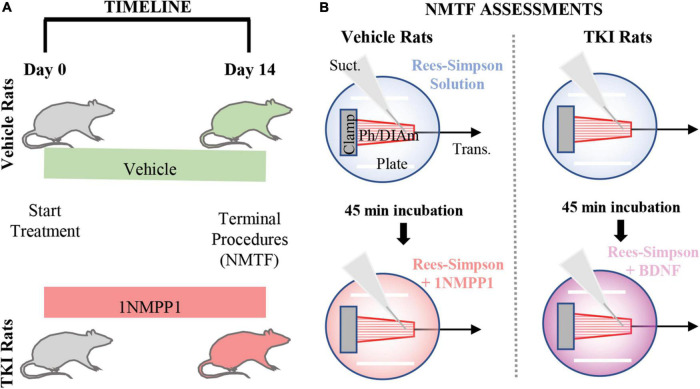
Experimental design and neuromuscular transmission failure (NMTF) assessment approach. **(A)** Timeline shows the order of the experiments, with vehicle and TKI groups randomly separated at day 0, when treatment with vehicle or 1NMPP1 commences. At day 14, terminal procedures (NMTF) are performed. **(B)** The NMTF assessment diagram shows the experimental setup of the Bulbring DIAm-phrenic nerve preparation (Ph/DIAm) clamped (Clamp) at the rib end and attached *via* suture to the transducer (*Trans.*). A suction electrode (Suct.) and platinum plate electrodes (Plate) stimulate the nerve and muscle, respectively. In both vehicle and TKI rats, NMTF is performed at 24°C in Rees-Simpson solution (transparent blue) bubbled with carbogen gas. Following the first NMTF trial, Ph/DIAm preps are pre-incubated for 45 min in either 1NMPP1 + Rees-Simpson solution (transparent red; vehicle rats) or BDNF + Rees-Simpson solution (transparent violet; TKI rats) prior to a second NMTF trial.

Two age-, weight-, and sex-matched groups were studied; DMSO (0.3%; vehicle) treated control *TrkB*^*F*616^ rats (Vehicle; *n* = 8) and 1NMPP1 (25 μM) treated *TrkB*^*F*616^ rats (TKI; *n* = 8). Treatment was initiated when the rats were ∼4 months of age (initial body weights – ∼240 g females and ∼530 g males) and continued for 14 days (Figure 1A).

While undergoing oral 1NMPP1 treatment, water intake was monitored daily. Intraperitoneal injections of 1NMPP1 (∼0.66 mg/kg) in *TrkB*^*F*616*A*^ mice have shown that TrkB inhibition occurs within an hour *in vivo* (Pareja-Cajiao et al., 2020). Based on the consumption in drinking water (25 μM), ∼1.4 mg/kg/day of 1NMPP1 is consumed by *TrkB*^*F*616*A*^ mice, similar to the 1.6 mg/kg/day consumed in the present study by our *TrB*^*F*616^ rats. The dosage in drinking water (25 μM) has previously been shown to be effective at reducing the ratio of phosphorylated TrkB protein to total TrkB protein in protein by ∼13-fold in the neural tissue of *TrkB*^*F*616*A*^ mice (Mantilla et al., 2014). We expect similar magnitudes of TrkB inhibition in *TrkB*^*F*616^ rats (i.e., >90%).

Treatment duration was based on previous demonstration of substantial DIAm NMTF following 7–14 days of altered synaptic activity in *TrkB*^*F*616*A*^ mice (Mantilla et al., 2014). At the terminal experiment, animals were deeply anesthetized by intramuscular injection of ketamine (90 mg/kg) and xylazine (10 mg/kg) and then exsanguinated.

### DIAm neuromuscular transmission

The left DIAm and phrenic nerve was excised and placed in a tissue bath (Bulbring, 1946) containing Rees-Simpson’s buffer (pH 7.4) at 26°C and gassed with carbogen (95 O_2_/5% CO_2_). A 2 mm wide segment of the costal DIAm was dissected with the phrenic nerve attached and placed in a tissue bath with the costal margin clamped and the central tendon attached to a calibrated force transducer (6,350, Cambridge Technology, Bedford, MA, USA). Optimal DIAm length for isometric force generation was set based on passive tension and maximal twitch responses. The muscle was directly stimulated with 0.5 ms-duration current pulses (701C, Aurora Scientific, Aurora, ON, Canada) using platinum plate electrodes placed on either side of the muscle. The phrenic nerve was stimulated *via* a suction electrode using 0.05 ms-duration current pulses. In both cases, stimulus strength was increased until maximal twitch responses were obtained and then set at 125% maximum (i.e., supramaximal, ∼140 mA for direct muscle and ∼25 mA for nerve stimulation). Thereafter, specific twitch and specific tetanic force responses to direct muscle stimulation at 40 Hz were calculated by normalizing force to the estimated cross-sectional area (CSA) of the DIAm strip (muscle CSA = muscle strip weight (g)/(Lo (cm) × 1.056 g/cm^3^) and expressed as N/cm^2^ (Johnson and Sieck, 1993). Evoked DIAm forces were digitized and recorded in LabChart software (ADI, Dunedin, New Zealand).

The procedure for estimating the extent of NMTF has been previously described in detail. Briefly, the phrenic nerve was repetitively stimulated at 40 Hz in 330-ms duration trains repeated each s (33% duty cycle) for a 120-s period. Every 15 s, the muscle was directly stimulated, and NMTF was assessed by comparing DIAm forces evoked by phrenic nerve vs. direct muscle stimulation (Aldrich et al., 1986; Kuei et al., 1990; Johnson and Sieck, 1993; van Lunteren et al., 2004; Personius and Sawyer, 2006; Rizzuto et al., 2015; Fogarty et al., 2019, 2020a). With NMTF, muscle fibers that are not activated by nerve stimulation due to NMTF are spared from muscle-derived contributions to fatigue (Sieck and Prakash, 1995). Accordingly, increased NMTF is reflected by greater differences between forces evoked by nerve compared to direct muscle stimulation (i.e., NMTF% is the % of force loss attributed to impaired neuromuscular transmission, not DIAm fatigue). The extent of NMTF was calculated using the following equation:


$$NMTF=\left[MF\text{/}MF_{init}–NF\text{/}NF_{init}\right]\text{/}MF\text{/}MF_{init}\;x\;\text{1}00)$$

Where MF = muscle force, MF_*init*_ = initial muscle force, NF = nerve force, and NF_*init*_ = initial nerve force, in a manner derived from the original quantitative attempts (Aldrich et al., 1986; Kuei et al., 1990). This formula measure the difference in nerve-derived fatigue compared to muscle-derived fatigue as a percentage of muscle-derived fatigue.

Intratrain fatigue was calculated by the difference between the maximum and minimum force within a given stimulus train (van Lunteren et al., 2004). Intratrain fatigue was assessed every 15 s for the duration of the NMTF procedure in all experimental groups.

We previously demonstrated an acute effect of 1NMPP1 on worsening NMTF in *TrkB*^*F*616*A*^ mice following 45 min of pre-incubation (Mantilla and Ermilov, 2012). In the vehicle group, following the first NMTF assessment, the tissue was incubated in 1NMPP1 (25 μM) for 45 min and subsequently, NMTF was measured a second time in the presence of 1NMPP1 (Figure 1B). In the TKI rat, following assessment of NMTF, a 45 min recovery period during which the preparation was bathed in BDNF (100 ng/ml; rhBDNF, R&D Systems, Minneapolis, MN, USA) was observed prior to testing NMTF in the presence of (Figure 1B). The peripheral effects of 1NMPP1 on BDNF/TrkB signaling activity occur locally with TrkB receptors at the pre-synaptic terminal (Gonzalez et al., 1999).

### DIAm neuromuscular junction morphology

Pre- and post-synaptic elements of NMJs at type-identified DIAm fibers were visualized using confocal microscopy, as previously described in detail (Prakash and Sieck, 1998; Mantilla et al., 2014; Fogarty et al., 2019, 2020a). Briefly, adjacent costal DIAm strips were fixed in 4% paraformaldehyde (PFA) and incubated with α-bungarotoxin conjugated to Alexa-Fluor 555 (0.1 μg/ml; B35451, Invitrogen Corp., Carlsbad, CA, USA) to label post-synaptic cholinergic receptors. An anti-synaptophysin antibody was used to label pre-synaptic terminals (1 mg/ml; sc17750, Santa Cruz Biotechnology Inc., Santa Cruz, CA, USA) with an Alexa-Fluor 647 conjugated donkey anti-mouse IgG secondary antibody (1:200; 715605159, Jackson Immuno Research Laboratories Inc., Baltimore, PA, USA) (Fogarty et al., 2015, 2019, 2020a). Confocal image stacks were obtained from NMJs visualized *en face* on the thoracic surface of the DIAm using a 60 x water immersion objective (1.3 NA), with 555 and 647 fluorophores imaged sequentially into a 1,200 × 1,200 xy pixel array in a *z*-stack series with a step of 2 μm. A mean of five NMJs (range: 3–9) were assessed per type per rat per group. Well-established morphological (size and complexity) criteria were used to classify NMJs at type I and IIa or type IIx and/or IIb DIAm fibers (Prakash and Sieck, 1998; Fogarty et al., 2019; Gonzalez Porras et al., 2019). Briefly, we defined NMJs with planar areas of <500 μm^2^ and on DIAm fibers of <50 μm diameters as being NMJs of type I and IIa fibers. Those NMJs with areas >500 μm^2^ on fibers >50 μm were considered type IIx and/or IIb NMJs. Where NMJs did not fit both criteria, they were excluded from type-specific analyses (11 NMJs in the present study), but retained for overall animal comparison estimates. Labeled axon terminals and motor endplates were circumscribed by a rectangular region of interest in Metamorph, and fluorescence intensities of α-bungarotoxin labeled motor endplates and synaptophysin labeled axon terminals were thresholded to generate binary images, and the extent of overlap was determined for each NMJ (Prakash and Sieck, 1998; Fogarty et al., 2019, 2020a). Briefy, in a projection image (∼6–12 z-slices), the area of pre-synaptic terminals that co-localized with post-synaptic endplate was divided by the total post-synaptic endplate area, and expressed as a %.

### Statistical analysis

The number of animals required to detect a biologically relevant difference of >20% (equivalent to prior studies of a similar mutant mouse) (Mantilla et al., 2014) was determined by power analysis (α = 0.05, β = 0.80) based on previous reports of the major outcome measure in rats [overall NMTF–mean (∼59.0) and standard deviation (∼8.5)] (Mantilla et al., 2004; Fogarty et al., 2019). Eight rats were deemed necessary for each group. Statistical analysis was performed using Prism 8 (Graphpad Software, San Diego, CA, USA) with two-way ANOVA and Bonferroni post-tests used to compare experimental groups and stimulation frequency. All omnibus F-values for all results are reported in Table 1, with the results section reporting specific Tukey or Bonferroni values for relevant comparisons. All data were assessed for normality with Shapiro-Wilk tests. *A priori* it was determined that within a particular data set, any data point outside two standard deviations from the mean was excluded from further analysis. Fortunately, we did not observe any outliers in the main outcome measures in the current study. Significance was set as *P* < 0.05, and all data are presented as means ± 95% confidence intervals (CI), unless otherwise stated. For the sake of clarity, acute vs. chronic effects of 1NMPP1 are reported separately in the results, despite statistical analyses (one- or two-way ANOVAs) being performed together in an omnibus fashion.

**TABLE 1:** One- and two-way ANOVA tables.

Parameter	ANOVA	F-value	*P*-value
Body mass (g)	two-way	Sex: F_(1,12)_ = 5.9 Group: F_(1,12)_ = 0.01	*P* = 0.03 *P* = 0.93
Initial NMTF (%)	one-way	F_(3,28)_ = 1.0	*P* = 0.39
120-s NMTF (%)	two-way	Group: F_(3,28)_ = 4.1 Time: F_(3,93)_ = 122.8	*P* = 0.015 *P* < 0.0001
Final NMTF (%)	one-way	F_(3,28)_ = 14.7	*P* < 0.0001
Intratrain fatigue (%)	two-way	Group: F_(3,28)_ = 16.1 Time: F_(3,93)_ = 83.8	*P* < 0.0001 *P* < 0.0001
NMJ apposition (%)	two-way	Group: F_(1,7)_ = 0.01 Fiber type: F_(1,7)_ = 1.8	*P* = 0.93 *P* = 0.81
NMJ relative planar area (%)	two-way	Group: F_(1,7)_ = 1.5 Fiber type: F_(1,7)_ = 20.1	*P* = 0.26 *P* = 0.009
NMJ pre-synaptic CSA (μm^2^)	two-way	Group: F_(1,7)_ = 0.4 Fiber type: F_(1,7)_ = 100.3	*P* = 0.54 *P* < 0.0001
NMJ endplate CSA (μm^2^)	two-way	Group: F_(1,7)_ = 0.6 Fiber type: F_(1,7)_ = 85.5	*P* = 0.48 *P* < 0.0001

Relative planar area is defined as the NMJ endplate CSA divided by NMJ orthogonal area (the rectangular *xy* boundaries of an endplate).

Sex was included as a biological variable in our experimental design; however, we previously found that there are no sex differences in the various rat DIAm (Fogarty et al., 2020b, 2021) and NMJ (Fogarty et al., 2019, 2020a; Gonzalez Porras et al., 2019) properties. Accordingly, data for females and males were combined for subsequent analyses.

## Results

### Chronic tropomyosin related kinase B kinase inhibition does not affect body mass

The initial and final body weights of TKI and vehicle treated rats were comparable, when sex differences were considered. As expected, the final body mass of females in both TKI (∼259 g, 7.5% increase) and vehicle (∼254 g, 5.4% increase) treated groups was significantly lower than males (TKI∼556 g, 3.7% increase and vehicle ∼540 g, 1% increase; *P* < 0.01; Two-way ANOVA; Table 1).

### Tropomyosin related kinase B kinase inhibition does not affect DIAm isometric specific force at 40 Hz muscle stimulation

**FIGURE 2:**
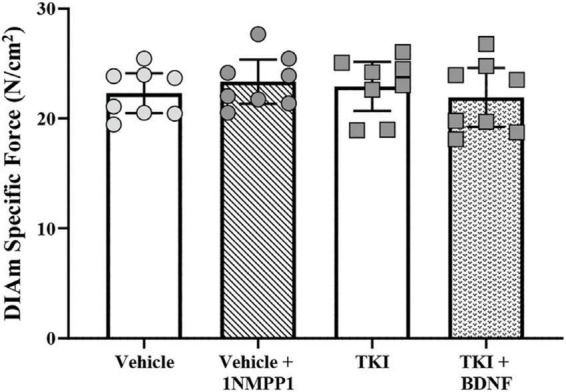
Unchanged isometric specific force in *TrkB*^*F*616^ rat DIAm treated acutely (in bath) or chronically (14 days) with 1NMPP1. Plot (mean ± 95% CI) illustrating increased final DIAm isometric specific force of vehicle + 1NMPP1, TKI and TKI + BDNF compared to vehicle rats (one-way ANOVA, Tukey’s post-test). *N* = 8 rats per group. Stimulations at 40 Hz reflect ∼80% of maximum.

Chronic TrkB kinase inhibition had no effect on DIAm isometric force generation. Isometric DIAm specific force evoked by 40 Hz direct muscle stimulation was unchanged in vehicle (22.3 ± 1.8 N/cm^2^), vehicle + 1NMPP1 (23.4 ± 2.1 N/cm^2^), TKI (22.9 ± 2.2 N/cm^2^) and TKI + BDNF (21.9 ± 2.7 N/cm^2^) rats (*P* = 0.71, one-way ANOVA; Figure 2).

### Acute and chronic tropomyosin related kinase B kinase inhibition worsens DIAm neuromuscular transmission failure

**FIGURE 3:**
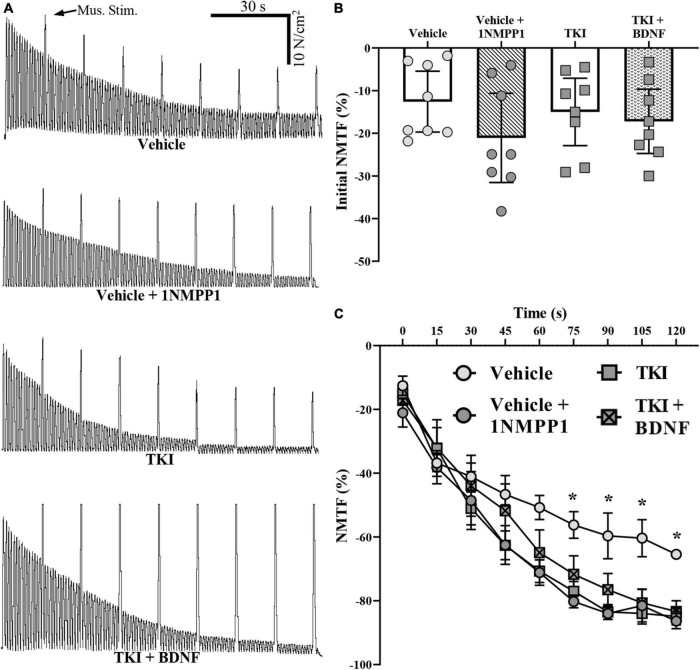
Neuromuscular transmission failure (NMTF) occurs more rapidly in *TrkB*^*F*616^ rat DIAm treated acutely (in bath) or chronically (14 days) with 1NMPP1. **(A)** Force traces of 120 s of NMTF inducing nerve stimulations, superimposed with direct muscle stimulation every 15 s of DIAm from control (vehicle), control following 45 min of pre-incubation with 1NMPP1 (vehicle + 1NMPP1, 14-day 1NMPP1-treated rats (TKI) and 14-day 1NMPP1-treated rats following 45 min of pre-incubation with BDNF (TKI + BDNF) rats. **(B)** Plot (mean ± 95% CI) illustrating unchanged initial DIAm NMTF in all groups (one-way ANOVA). **(C)** Plot (mean ± SEM) of time course of NMTF illustrating increased failure of vehicle + 1NMPP1, TKI and TKI + BDNF compared to vehicle rats (denoted by *). *P < 0.05.

We assessed the nerve and muscle contributions to DIAm force loss across a 120 s period of stimulations in TKI and vehicle treated rats (Figure 3). Compared to direct muscle stimulation, the initial DIAm force evoked by 40 Hz phrenic nerve stimulation was less in both experimental groups (Figure 3). These failures may be due to pre-synaptic axon propagation/branch point failure, pre-synatic release failures (reduced quantal content, reduced recruitment to active zones from the readily releasable pool) and or desensitization of the acetylcholine receptors (Smith, 1979, 1980; Sieck and Prakash, 1995; Davis et al., 2022). The initial difference in DIAm force evoked by nerve compared to muscle was similar in the TKI and vehicle groups (*P* = 0.39, one-way ANOVA; Figure 3B), with forces evoked by phrenic nerve stimulation being 15.0 ± 7.9% lower in the TKI group, compared to 12.6 ± 7.1% lower in the vehicle group and 20.5 ± 9.9% (*P* = 0.96) in the vehicle + 1NMPP1 group (*P* = 0.96; Tukey’s post-test; Table 1; Figure 3B).

During repetitive stimulation across the 120-s period, the forces evoked by nerve stimulation declined to a much greater extent than those evoked by direct muscle stimulation in both groups reflecting NMTF (Figure 3A). The extent of DIAm NMTF was significantly worsened in the TKI group compared to vehicle treated controls, reflecting an effect of chronic TrkB kinase inhibition and time (Two-way ANOVA; Table 1; Figure 3C). Beginning at 60 s of nerve stimulation, the extent of NMTF was exaggerated in the TKI group and the vehicle + 1NMPP1 group compared to vehicle controls (*P* < 0.05 for all time comparisons of TKI and vehicle from 60-s, Bonferroni post-tests; Table 1; Figure 3C).

**FIGURE 4:**
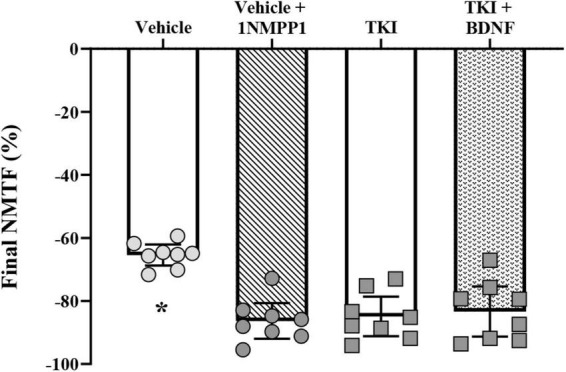
Increased final neuromuscular transmission failure (NMTF) in *TrkB*^*F*616^ rat DIAm treated acutely (in bath) or chronically (14 days) with 1NMPP1. Plot (mean ± 95% CI) illustrating increased final DIAm NMTF of vehicle + 1NMPP1, TKI and TKI + BDNF compared to vehicle rats (one-way ANOVA, Tukey’s post-test). *N* = 8 rats per group. In all cases *P < 0.05, compared to vehicle.

After 120 s of nerve stimulation, the final extent of NMTF was worsened by ∼one-third in rats with chronically inhibited TrkB kinase activity (TKI: −84.9 ± 6.3%) compared to vehicle treated control rats (vehicle: −65.4 ± 3.4%; *P* < 0.0001, Tukey’s post-test; Table 1; Figure 4). Compared to vehicle controls, acute 1NMPP1 worsened NMTF by ∼one-third (vehicle + 1NMPP1: −86.4 ± 5.6%; *P* < 0.0001, Tukey’s post-test; Table 1; Figure 4).

### Acute brain-derived neurotrophic factor does not rescue DIAm neuromuscular transmission failure in rats with chronic tropomyosin related kinase B kinase inhibition

In rats with chronic TrkB kinase inhibition (TKI group), acute BDNF did not improve initial NMTF (i.e., the% of failure during the first stimulus train), with differences in initial muscle vs. nerve-evoked forces equivalent between TKI + BDNF (−15.0 ± 7.9%) and TKI (−17.2 ± 7.5%) groups (*P* = 0.99, Tukey’s post-test; Table 1, Figure 3B).

Acute BDNF treatment did not rescue the progressive worsening of DIAm NMTF in rats with chronic TrkB kinase inhibition during the 120 s of repetitive nerve stimulation. The progression of NMTF followed a similar time course in both TKI + BDNF and TKI groups (*P* > 0.99 for all time comparisons, Bonferroni post-test; Table 1, Figure 3C).

Acute BDNF did not rescue NMTF after 120 s of nerve stimulation in rats with chronic TrkB kinase inhibition, with final NMTF equivalent between TKI + BDNF (−83.4 ± 8.0%) and TKI (−84.9 ± 6.3%) groups (*P* > 0.99, Tukey’s post-test; Table 1, Figure 4).

### Intratrain fatigue was greater with acute and chronic tropomyosin related kinase B kinase inhibition and not rescued by acute brain-derived neurotrophic factor

**FIGURE 5:**
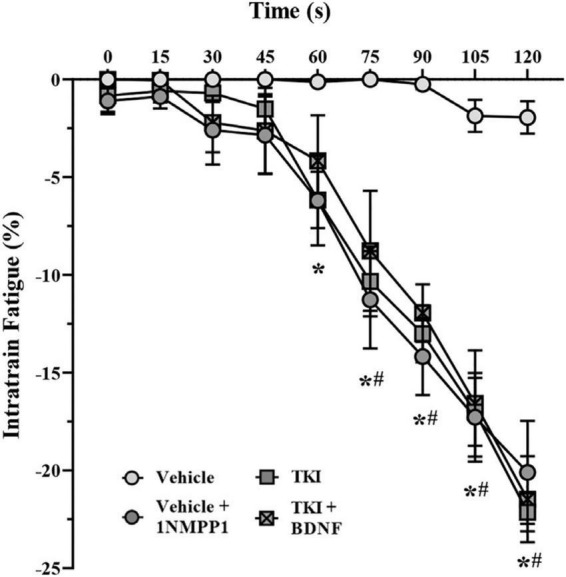
Intratrain fatigue occurs more rapidly in *TrkB*^*F*616^ rat DIAm treated acutely (in bath) or chronically (14 days) with 1NMPP1. Plot (mean ± SEM) of time course of intratrain faatigue illustrating increased failure of vehicle + 1NMPP1 (denoted by ^#^), TKI (denoted by *), and TKI + BDNF (denoted by ^#^) compared to vehicle rats (two-way ANOVA, Bonferroni post-tests). *N* = 8 rats per group. In all cases *^#^*P* < 0.05, compared to vehicle.

The intratrain fatigue assessed the degree of force loss within a train of stimulation. During repetitive stimulation across the 120-s period, the intratrain fatigue was negligible in vehicle control rats, while progressively increasing from 60 s TKI rats (*P* < 0.05) and from 75 s in vehicle + 1NMPP1 (*P* < 0.05) and TKI + BDNF (*P* < 0.05) rats (two-way ANOVA; Table 1; Figure 5). By the conclusion of the 120-s period, the intratrain fatigue was negligible in vehicle control rats (−1.9 ± 1.5%), and significantly increased in vehicle + 1NMPP1 (−20.1 ± 6.3%), TKI (–22.1 ± −2.3 N/cm^2^) and TKI + BDNF (21.5 ± 5.2 N/cm^2^) rats (Figure 5).

### Chronic tropomyosin related kinase B kinase inhibition did not change neuromuscular junction morphology

**FIGURE 6:**
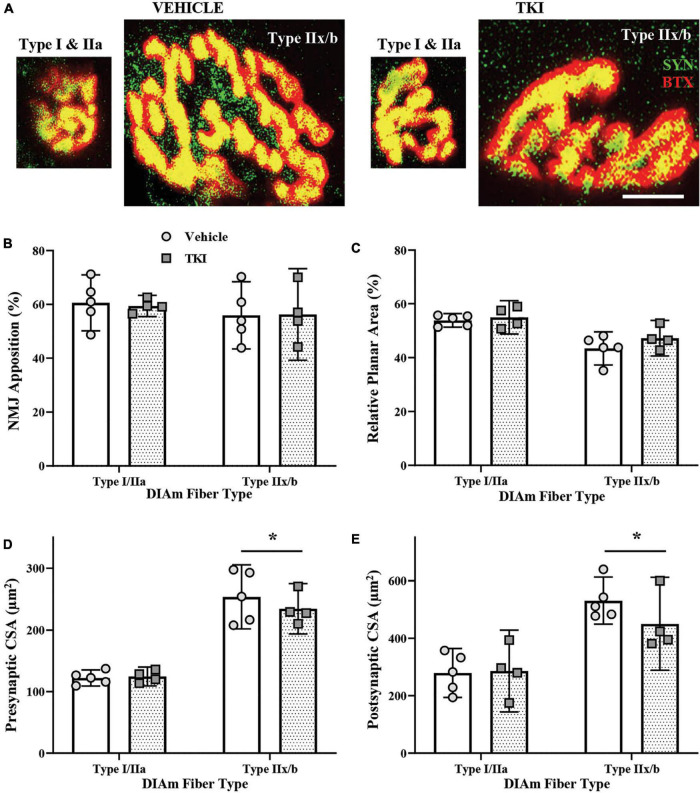
Neuromuscular junction (NMJ) morphology is conserved in *TrkB*^*F*616^ rats treated with 1NMPP1 for 14 days. **(A)** Pictomicrographs show NMJs labeled with pre-synaptic Synaptophysin (SYN-green) and post-synaptic α-Bungarotoxin (BTX-red) from type I or IIa fibers and type IIx and/or IIb DIAm fibers in vehicle control rats (left pair) and TKI rats (right pair) following 14 days of 1NMPP1 treatment. **(B)** Plot (mean ± 95% CI) shows mean NMJ pre- and post-synaptic apposition (%) is unchanged between vehicle and TKI rats. **(C)** Plot (mean ± 95% CI) shows mean NMJ relative planar area (%) is unchanged between vehicle and TKI rats. **(D)** Plot (mean ± 95% CI) shows mean NMJ pre-synaptic CSA (μm^2^) is unchanged between vehicle and TKI rats, with CSA in type IIx and/or IIb fibers larger than type I or IIa fibers, regardless of treatment. **(E)** Plot (mean ± 95% CI) shows mean NMJ endplate CSA (μm^2^) is unchanged between vehicle and TKI rats, with CSA in type IIx and/or IIb fibers larger than type I or IIa fibers, regardless of treatment. Two-Way ANOVAs with Bonferroni post-tests; *n* = 5 vehicle, *n* = 4 TKI rats. *Indicates difference between type I or IIa fibers and type IIx and/or IIb fibers, *P* < 0.05. Scalebar = 10 μm.

Neuromuscular junction were evaluated using α-bungarotoxin labeled motor endplates and synaptophysin labeled axon terminals (Figure 6). The gross innervation of DIAm NMJs was assessed using apposition estimates of how much post-synaptic area was occupied by pre-synaptic terminals (Figure 6B). The mean NMJ apposition of type I and IIa fibers (∼60%) or type IIx and/or IIb (∼56%) DIAm fibers was not different between vehicle and TKI groups, nor dependent of fiber type (Two-way ANOVA; Table 1, Figure 6B). In addition, we did not observe any difference in apposition (%) of individual NMJs between vehicle (60.7 ± 5.1%) and TKI rats (59.4 ± 6.0%; *P* = 0.75, Student’s unpaired *t*-test).

The complexity of DIAm NMJs was estimated using relative planar area measurements (NMJ endplate CSA divided by NMJ orthogonal area, with orthogonal areas defined by a rectangle that encompasses the *xy* boundaries of an endplate) (Mantilla et al., 2014). The mean relative planar area of type I and IIa fibers (∼45%) or type IIx and/or IIb (∼50%) DIAm fibers was not different between vehicle and TKI groups, nor dependent of fiber type (Two-way ANOVA; Table 1, Figure 6C). In addition, we did not observe any difference in apposition (%) of individual NMJs between vehicle (48.7 ± 4.2%) and TKI rats (49.4 ± 3.4%; *P* = 0.61, Student’s unpaired *t*-test).

Neuromuscular junction pre-synaptic terminal was assessed using overall terminal CSA. The mean terminal CSA of type I and IIa fibers (∼123 μm^2^) or type IIx and/or IIb (∼240 μm^2^) DIAm fibers was not different between vehicle and TKI groups, nor dependent of fiber type (Two-way ANOVA; Table 1, Figure 6D). In both vehicle (*P* = 0.0002) and TKI rats (*P* = 0.001), type IIx and/or IIb pre-synaptic terminal CSAs was larger than I and IIa (Bonferroni *post-hoc* tests; Figure 6D). In addition, we did not observe any difference in endplate CSA of individual NMJs between vehicle (182 ± 35 μm^2^) and TKI rats (184 ± 24 μm^2^; *P* = 0.93, Student’s unpaired *t*-test).

Neuromuscular junction endplates was assessed using overall endplate CSA. The mean endplate CSA of type I and IIa fibers (∼280 μm^2^) or type IIx and/or IIb (∼500 μm^2^) DIAm fibers was not different between vehicle and TKI groups, nor dependent of fiber type (Two-way ANOVA; Table 1, Figure 6E). In both vehicle (*P* < 0.0001) and TKI rats (*P* = 0.004), type IIx and/or IIb endplates CSAs were larger than I and IIa (Bonferroni *post-hoc* tests; Figure 6E). In addition, we did not observe any difference in endplate CSA of individual NMJs between vehicle (417 ± 63 μm^2^) and TKI rats (386 ± 49 μm^2^; *P* = 0.42, Student’s unpaired *t*-test).

## Discussion

The results of the present study demonstrate three main findings in rats with inhibition of BDNF/TrkB signaling: (i) DIAm neuromuscular transmission is markedly impaired in *TrkB*^*F*616^ rats treated with 1NMPP1 for 14 days (TKI rats), compared to vehicle controls; (ii) DIAm neuromuscular transmission is also markedly impaired in *TrkB*^*F*616^ rats with acute pre-incubation with 1NMPP1 (vehicle + 1NMPP1 rats), compared to vehicle rats without 1NMPP1; and (iii) gross morphological apposition of pre- and post-synaptic elements of DIAm NMJ is maintained in TKI rats, without any evidence of endplate expansion. Taken together, these results support the hypothesis that BDNF/TrkB signaling has a potent effect on DIAm neuromuscular transmission. Importantly in rodents, absence of BDNF/TrkB signaling does not reduce the intrinsic contractile and fatigue properties of DIAm (Mantilla and Ermilov, 2012; Mantilla et al., 2014; Greising et al., 2015a), nor does increased neurotrophin support enhance force generation (Mantilla et al., 2004; Mantilla and Ermilov, 2012). These finding are consistent with the absence of any changes in isometric specific force (∼22 N/cm^2^) during 40 Hz stimulations in the present study. Notably, although 40 Hz stimulations are tetanic, they are sub-maximal and ∼80% of the maximum observed in young adult rats (Fogarty et al., 2020b). Due to the pre-synaptic nature of BDNF/TrkB signaling (Lohof et al., 1993; Garcia et al., 2010b), it is highly unlikely that altered BDNF/TrkB signaling shifts the force-frequency relationship, though this was not directly tested. Thus, the remainder of our study focused on the neuromuscular transmission and the NMJ.

In the past, we have characterized NMTF with respect to initial failures and failures across the 120 s stimulation period. Excess NMTF may be caused by propagation failure (axon blockade or branch point failures), failure of synaptic vesicle release or release and vesicle recruitment kinetics, receptor desensitization or derangement in the apposition of pre-, post-synaptic or lamellar structures of the NMJ (Sieck and Prakash, 1995). These neurotransmission failures are more apparent at higher stimulation frequencies (i.e., >35 Hz), compared to lower frequencies (<20 Hz) (Fournier et al., 1991; Johnson and Sieck, 1993; Personius and Sawyer, 2006; Rizzuto et al., 2015; Fogarty et al., 2019, 2020a) and we chose 40 Hz to directly compare to prior studies in *TrkB*^*F*616*A*^ mice (Table 1; Mantilla and Ermilov, 2012; Mantilla et al., 2014; Greising et al., 2015a), prevent any increase in axonal propagation failure at >75 Hz stimulation (Burke et al., 1971, 1973; Clamann and Robinson, 1985; Sandercock et al., 1985; Johnson and Sieck, 1993; Sieck and Prakash, 1995), and simplify the experimental design by removing the need for a pulse control group, where duty cycle is altered to keep the overall pulse numbers identical between stimulation frequencies (Fogarty et al., 2019, 2020a). In preparations from young healthy rodents, initial DIAm NMTF is ∼15% (Johnson and Sieck, 1993; Fogarty et al., 2019, 2020a). In the current study, we show that neither acute nor chronic inhibition of BDNF/TrkB signaling influences initial DIAm NMTF, similar to past studies in the TrkB^*F*616*A*^ mouse (Mantilla et al., 2014). By contrast, acute and chronic inhibition of BDNF/TrkB signaling increased NMTF considerably during repetitive stimulation when compared to vehicle controls. This worsening of DIAm NMTF was similar to observations in *TrkB*^*F*616*A*^ mice treated acutely [30 min being sufficient for an effect of 1NMPP1 (10 mM) in the tissue bath] (Mantilla and Ermilov, 2012) or chronically with 1NMPP1 in drinking water (25 μM) (Mantilla et al., 2014; Greising et al., 2015a). A full accounting of the similarities and differences between the *TrkB*^*F*616*A*^ mouse and the *TrkB*^*F*616^ rat is provided in Table 2. Notably, we show acute BDNF was unable to rescue NMTF in rats with chronic (14 day) TrkB kinase inhibition, entirely consistent with the effects of BDNF/TrkB signaling *via* high-affinity TrKB_FL_ receptors. In support, similar findings to the present study are found when BDNF/TrkB signaling is diminished in the presence of TrkB kinase inhibitor K252a (Mantilla et al., 2004). We do not consider BDNF concentrations used in the present study to be below that required to see improved neuromuscular transmission, as we have shown 100 ng/ml is sufficient to decrease NMTF in adult rats (Mantilla et al., 2004) and is consistent with physiological levels (Berninger et al., 1993; Jovanovic et al., 2000). However, whether or not increased BDNF concentration beyond physiological levels has an effect on rats where BDNF/TrkB signaling is acutely or chronically inhibited was not assessed. Likewise, diffusion into the Bulbring phrenic nerve-DIAm preparation at non-physiologic temperatures (i.e., 26°C) is also not a likely source of BDNF ineffectiveness in *TrkB*^*F*616^ rats, as prior studies of exogenous BDNF tissue bath application show effective enhancement of neurotransmission.

**TABLE 2:** Comparison of DIAm properties of *TrkB*^*F*616^ rats and *TrkB*^*F*616*A*^ mice.

Parameter	Rat	Mouse	*TrkB*^*F*616*A*^ mouse references
Specific force	Unchanged	Unchanged	(Mantilla and Ermilov, 2012; Mantilla et al., 2014; Greising et al., 2015a)
Fatigue index	NA	Unchanged	(Mantilla and Ermilov, 2012; Mantilla et al., 2014)
Pdi_max_	NA	Reduced	(Pareja-Cajiao et al., 2020)
Initial NMTF (Acute)	Unchanged	Unchanged	(Greising et al., 2015a)
Initial NMTF (Chronic)	Unchanged	Unchanged	(Mantilla et al., 2014)
Final NMTF (Acute)	Increased Failure	Increased Failure	(Mantilla and Ermilov, 2012; Greising et al., 2015a)
Final NMTF (Chronic)	Increased Failure	Increased Failure	(Mantilla et al., 2014)
TrkB agonist improves NMTF	No	No	(Mantilla and Ermilov, 2012; Greising et al., 2017)
Intratrain fatigue	Increased	NA	
NMJ apposition	Unchanged	Increased	(Mantilla et al., 2014; Greising et al., 2015b)
NMJ apposition (Stratified)	Unchanged	NA	
NMJ relative planar area	Unchanged	Increased	(Mantilla et al., 2014)
NMJ relative planar area (Stratified)	Unchanged	NA	
Pre-synaptic terminal	Unchanged	Unchanged Reduced	(Mantilla et al., 2014) (Greising et al., 2015b)
Post-synaptic endplate	Unchanged	Unchanged	(Mantilla et al., 2014; Greising et al., 2015b)
mEPP amplitude	NA	Increased	(Mantilla et al., 2014)
EPP amplitude	NA	Increased	(Mantilla et al., 2014)
Quantal content	NA	Unchanged	(Mantilla et al., 2014)

Acute effects are limited to <1 h, chronic effects limited to in vivo treatment >7 days. Stratified refers to assessments done distinguishing between type I and IIa DIAm fibers and type IIx/IIb DIAm fibers. EPP, evoked endplate potential; mEPP, miniature endplate potential; NA, not assessed; Pdi_max_, maximum *trans*-diaphragmatic pressure.

Identifying the mechanism of NMTF induced by impaired BDNF/TrkB signaling was not the focus of this investigation, although our investigation uncovered clues as to where the locus of deficit occurs. It is unlikely that the acute 1NMPP1 inhibition TrkB alters the number of axon branch points, although if this did occur in the chronic scenario, we would expect differences in the magnitude of NMTF between acute and chronic studies. Indeed, deletion of TrkB during development results in excessive developmental motor neuron death (Klein et al., 1993; Klein, 1994), and subsequent increase in muscle innervation ratio (i.e., greater branching of individual motor neurons in order to innervate all muscle fibers of a particular motor unit group). We are currently investigating whether impaired BDNF/TrkB signaling in otherwise healthy rats affects phrenic motor neuron numbers and thus innervation ratios. If impaired BDNF/TrkB signaling leads to frank motor neuron death, increased NMTF compared to the acute scenario would be expected, with additional branch point failures on top of pre-synaptic release/vesicle recruitment impairments. Based on the present study, motor neuron death could be related to differences in long vs. short-term actions of BDNF/TrkB signaling–with long-term activation related to gene transcription factor alterations and neural survival, *via* CREB (Finkbeiner et al., 1997) and short-term actions related to synaptic transmission (Mantilla et al., 2004; Ermilov et al., 2007).

Other investigators have used intratrain fatigue to assess neuromuscular transmission in rodent models of neuromotor disorders such as myasthenia gravis (Personius and Sawyer, 2006) and amyotrophic lateral sclerosis (Rizzuto et al., 2015). Notably, these models *mdx* and *SOD1^*G*93*A*^* mice, respectively, exhibit gross disturbances in NMJ morphology (Gurney et al., 1994; Steyn et al., 2013; Pratt et al., 2015), with older *mdx* mice having marked DIAm ventilatory phenotype during awake states (de Zelicourt et al., 2022)–indicating effects on DIAm that are not selective for particular motor unit types (Davis et al., 2022). By contrast, amyotrophic sclerosis is associated with a high degree of motor unit type-specific motor neuron degeneration and death (Kiernan and Hudson, 1991; Nijssen et al., 2017; Fogarty, 2018; Ding et al., 2022), likely increasing axon propagation/branch point failure contributions to intratrain fatigue and NMTF, in addition to the contributions of denervated NMJs. The magnitude of the increased intratrain fatigue observed in *mdx* (∼30%) and *SOD1^*G*93*A*^* mice (∼30%) was greater than that observed in the present study (∼20%), suggesting that disorders of NMJ structural integrity are not as salient a feature in *TrkB*^*F*616^ rats as in myasthenia gravis or amyotrophic lateral sclerosis. Regardless, intratrain fatigue assessment, similar to NMTF assessment includes the time delay of excitation-contraction coupling, making assessments of neurotransmission at NMJs necessary to determine in detail acetylcholine release and vesicle recruitment kinetics (Rowley et al., 2007). These delay factors include the period between muscle action potential, sarcolemmal Ca^2+^ release, binding of Ca^2+^ to troponin and the actin and myosin interactions resulting in muscle shortening. Assessing miniature and evoked endplate potentials (mEPP and EPP, respectively) without muscle contraction (e.g., with conotoxin) would remove these impediments to interpretation regarding acetylcholine release kinetics and sensitivity.

Gross morphology of pre- and post-synaptic elements of NMJs was unchanged with chronic TrkB inhibition. Thus, it is likely that altered neurotransmitter release at pre-synaptic terminals (quantal content and non-uniformity of release) (Bennett and Lavidis, 1989; Rowley et al., 2007) or excessive post-synaptic desensitization of acetylcholine receptors, which also contributes to decreased force (Katz and Thesleff, 1957a; Sakmann et al., 1980), were the likely cause of increased NMTF following both acute or chronic inhibition of BDNF/TrkB signaling. Previously, we found that the effect of BDNF/TrkB signaling at DIAm NMJs is most likely due to pre-synaptic mechanisms, since there are no effects of BDNF/TrkB inhibition on DIAm force and fatigue properties (Mantilla et al., 2004) and desensitization of the acetylcholine receptor requiring prolonged non-physiological activation or receptor antagonists is necessary for this to occur (Katz and Thesleff, 1957b; Davis et al., 2022). In support, repetitive activations of DIAm during ventilation are not affected by acute administration of 1NMPP1 in *TrkB*^*F*616*A*^ mice (Pareja-Cajiao et al., 2020). Furthermore, acute BDNF/TrkB signaling has been shown to enhance quantal content at DIAm NMJs (Ermilov et al., 2007). Future investigation of quantal content, axon terminal vesicle density, endplate potentials and differences in vesicle kinetics or recruitment of vesicles to the readily-releasable pool may quantify the contribution of specific pre-synaptic disturbances to the NMTF phenotype in *TrkB*^*F*616^ rats.

Our results show NMJ morphological findings inconsistent with gross NMJ derangement being a contributing factor to worsened neuromuscular transmission in *TrkB*^*F*616^ rats. Indeed, the rapidity of the effect of acute 1NMPP1 in worsening NMTF almost precludes an effect of BDNF/TrkB signaling on pre- and post-synaptic apposition. In other mice that are deficient in TrkB from embryogenesis (i.e., *TrkB*^±^ mice), morphometric changes of NMJs were sufficient to impair neuromuscular transmission (Kulakowski et al., 2011). In the present study, the extent of pre- and post-synaptic apposition was consistent with our other reports in young healthy rat DIAm (Prakash and Sieck, 1998; Fogarty et al., 2019; Gonzalez Porras et al., 2019). Although our past reports found morphological NMJ disruptions following chronic TrkB kinase inhibition in *TrkBF^616*A*^* mice (Mantilla et al., 2014; Greising et al., 2015b), the magnitude of these changes was modest or inconsistent (Mantilla et al., 2014; Greising et al., 2015b), and fiber type differences were not accounted for (see Table 2). Moreover, reduced apposition, when present, is not necessarily associated with a decline in the efficacy of synaptic transmission (Willadt et al., 2016) and neither is increased NMTF inevitably associated with NMJ impairments (Fogarty et al., 2020a; Davis et al., 2022). There are some suggestions of altered endplate CSA in various conditions, including aging (Bao et al., 2020). In a particularly relevant study, it was reported in soleus NMJs that acetylcholine receptor (endplate) CSA (labeled with CTB, as here) increased with age (between 12 and 24 months)–with concomitant reduction in TrkB (Personius and Parker, 2013). Notably in the present study, we did not observe any endplate expansion, although our time period was of much shorter duration (14 days). Although pre- and post-synaptic elements of the NMJ have been morphologically characterized in many developmental and disease contexts, remarkably little has been reported regarding the effects of diminished BDNF/TrkB signaling on basal lamina (Knight et al., 2003; Chand et al., 2015). Impairments in the basal lamina are known to contribute to increased DIAm neuromuscular deficits in aging (Lee et al., 2017); however, fiber type specific information regarding basal lamina of DIAm NMJs is lacking. In future studies, probing of NMJ ultrastructure for differences in the number of readily releasable pool vesicles, recycling pool vesicles, and reserve pool vesicles (Wilson, 1979; Rizzoli and Betz, 2005; Tabares et al., 2007; Wyatt and Balice-Gordon, 2008) may yield insights into any anatomical basis for functional neurotransmission impairments in response to chronic BDNF/TrkB signaling inhibition.

In summary, we provide evidence that inhibition of BDNF/TrkB signaling in *TrkB*^*F*616^ rats results in marked DIAm neuromuscular transmission deficits, in the absence of gross perturbations to NMJ morphology. We are confident that the *TrkB*^*F*616^ rat model will be highly useful for examining the effects of BDNF/TrkB signaling inhibition on motor function in various scenarios, particularly aging and cervical spinal cord injury.

## Data availability statement

The original contributions presented in this study are included in the article/supplementary material, further inquiries can be directed to the corresponding author.

## Ethics statement

This animal study was reviewed and approved by Institutional Animal Care and Use Committee (IACUC) at Mayo Clinic.

## Author contributions

MF and GS conceived the rationale and designed the study. MF and OK performed experiments and analyzed the data. MF wrote the first draft of the manuscript. MF, OK, and GS made the figures. All authors revised the manuscript, read, and approved the submitted version.
